# The pain colour of children with toothache in Turkish population

**DOI:** 10.1186/s12903-019-0756-y

**Published:** 2019-04-18

**Authors:** Halenur Altan, Hüseyin Çevik, Serkan Doğru, Alem Coşgun, Mustafa Süren, İsmail Okan

**Affiliations:** 1 Department of Pediatric Dentistry, Gaziosmanpaşa University, Faculty of Dentistry, Tokat, Turkey; 2 Department of Anesthesiology and Reanimation, Gaziosmanpaşa University, Faculty of Medicine, Tokat, Turkey; 3 Kayseri Nimet Bayraktar Oral and Dental Health Hospital, Clinic of Pediatric Dentistry, Kayseri, Turkey; 4 Department of General Surgery, Gaziosmanpaşa University, Faculty of Medicine, Tokat, Turkey

**Keywords:** Children, Colour, Pain assessment, Toothache

## Abstract

**Background:**

Toothache is a common consequence of untreated caries, predisposed by poor oral hygiene and high caries risk. Most children expressed their pain through their parents or carers. The aim of this study was to determine the colour of pain presence and absence.

**Methods:**

Patients aged between 4 and 14 and referred to a dentist for the first time due to toothache had a short-term pain of 1 month caused by deep cavities. The children chose paintings from the box of 24 standard colours (Crayola, Spain) and the circles were painted. Pain was rated by children on the Visual Analoge Scale. Normality and variance were tested using the one-sample Kolmogorov-Smirnov test. Associations were performed by using the Pearson correlation coefficient. Analyses were completed by using the Statistical Package for Social Sciences (SPSS Inc., Chicago, IL) version 20.0 program.

**Results:**

A total of 147 patients including 78 girls (53.1%) and 69 boys (46.9%) were included in the study. The principal component analysis showed that red has the highest factor loading in children with pain, whereas yellow was the other highest one in children without pain.

**Conclusion:**

The presence of pain was mainly associated with red, and the absence of pain was associated with yellow in Turkish population. Description of pain with colour can be useful tool to recognize the children and to improve dentist-patient or dentist-parents communication.

## Background

Pain is an unpleasant sensory and emotional experience associated with actual or potential tissue damage, or described in terms of such damage [1]. Toothache, stomachache, headache, limb pain, and chest or back pain are experienced occasionally or frequently by up to 30% of children. Toothache is consistently associated with the population’s levels of caries risk factor, and it is highly prevalent among children [2]. Dental pain in children and adolescents affects community health, individuals and their families by 15–25%. The frequency and severity of pain negatively affects daily activities such as nutrition, sleep, inability to go to school, and smiling and oral care [3].

The presence and intensity of the pain is reported by the patient himself, and this way is the gold standart [4]. The lack of children’s pain experiences and the low number of words through which they can express their problems make it difficult for children to correctly identify their pain [5]. For this reason, unlike adults, most children expressed their pain through their parents or carers [4, 6].

Various methods of evaluation have been developed for the expression of pain by children. These methods vary with the change in the child’s ability to perceive, interpret, and express in relation to developmental stages, past pain experiences, and other environmental factors [7–9]. There are various scales to describe the intensity of pain. These include pain measure scales (visual analog scale, numerical scale, face scales, colour scale) based on personal expression; pain measure scales based on behavioral patterns, combined scales (Children’s Hospital of Easter of Ontario Pain Scale (CHEOPS)) and physiological changes (blood pressure, pulse, respiration, oxygen saturation) [10].

Children’s capability of using self-reporting pain tools changes over time [11]. Children under three years of age are unable to quantify pain using the currently available self-report measures. Children over the age of three can rate their pain on appropriate scales [4, 12]. For younger children with undeveloped numerical concept knowledge, an alternative means for communicating their pain is provided by colour identification [11].

Several colour-based tools have been developed for the assessment of pain [4–7, 13, 14]. The Analogic Chromatic Continuous Scale is composed of a color scale, from light pink (no pain) through shades of red to black (severe pain). The colour Analog Scale is a similar scale which was developed to be used in pediatric populations and includes the respondent sliding a marker along a colour thermometer from light pink (no pain) to deep red (worst pain) [14]. The pictorial Scale of Pain Intensity is composed of a sequence of vertically aligned red circles, which increase in size to represent increasing pain intensity [15]. The PAULA pain-meter is composed of five colored emoticon faces, with the red colored face representing the worst imaginable and the yellow-colored face representing no pain [16, 17].

Correlation between the prevalence of dental caries and toothache has been reported to be moderate to strong [3]. Toothache is a common consequence of untreated caries occurring due to poor oral hygiene, high caries activity or excessive use of refined food [3]. Children in Turkey have the highest caries risk among children in European countries, and hence, pain (toothache) experience begins at younger ages [18]. The aim of this study was to examine whether they could use colour to describe the pain of children experiencing with toothache and show the any relation between pain intensity and pain colour.

## Methods

### Sample

Children admitted to the outpatient unit of the Department of Pediatric Dentistry, Gaziosmanpasa University, who had acute toothache, were invited to participate in the study. Patients with no systemic diseases, aged between 4 and 14, referred to a dentist for the first time due to toothache with a short-term pain of up to 1 month caused by deep cavities in teeth were included in the study.

Children were excluded if they had altered mental status or any degree of cognitive impairment, were non-cooperative, had color-blindness or motor abnormalities leading to score of the scales, could not understand explanations and commands in Turkish, or response time or colour selection lasting more than ten minutes. Children who had no pain, and experienced any dental or facial prolonged pain for more than one month were excluded from the study.

**Fig. 1:**
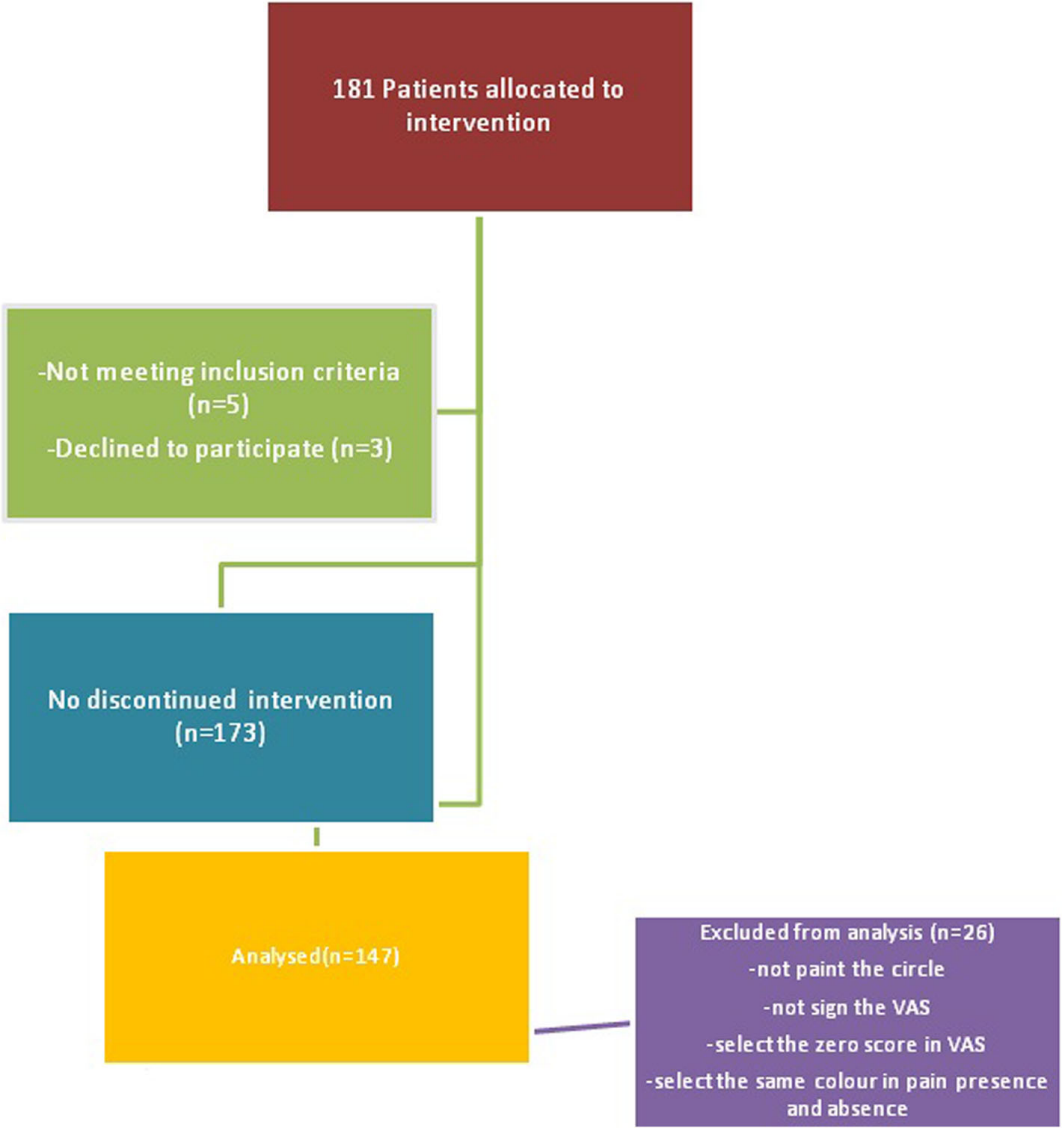
Consort Flow Diagram

One hundred eighty-one pediatric patients were included in the study and a total of 147 patients were evaluated in accordance with the exclusion criteria (Fig. 1).

### Study design

This is a prospective descriptive study. The study protocol was approved by the Gaziosmanpaşa University Clinical Research Local Ethics Committee (17- KAEK-070). Written consent was obtained from parents.

### Colours and pain

**Fig. 2:**
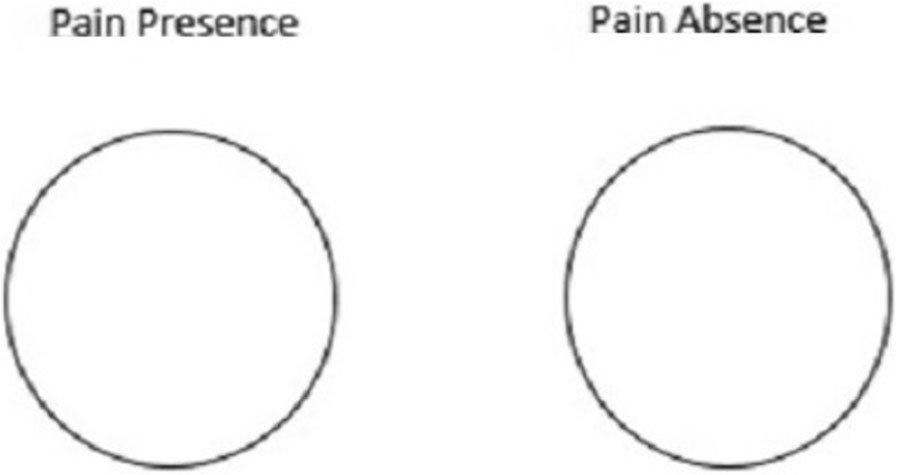
Circles for painting in pain absence and presence

Two circles were drawn on A5 size white paper entitled “Pain Presence” and “Pain Absence” (Fig. 2). We asked “Which colour do you want to use if you have pain?” and “Which colour do you want to use if you have no pain?” The children chose paintings from the box of 24 standard colours (Crayola, Spain) and the circles were painted.

### Visual pain scale

**Fig. 3:**
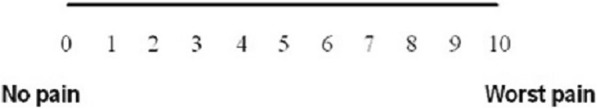
Visual Analoge Scale

When the painting was completed children rated their pain on the Visual Analog Scale (Fig. 3).

### Statistical analysis

We would detect a simple correlation (r = 0.3) between VAS and pain presence using a two-sided test with an alpha of 0.05 and a power of 80%, the required sample size is approximate 113 (*n* = 113).

The Kaiser-Meyer-Olkin measure of sampling adequacy and Bartlett’s test of sphericity were performed to determine the evaluation of the factor analysis for colours. The interaction between colours and pain intensity was completed by using the principal components analysis. To perform this analysis, the components were extracted with an eigen value of higher than one. Varimax rotation with Kaiser normalization was chosen, and the rotation converged in seven iterations, with values below 0.40 being excluded.

The distribution of the variables were analyzed using one-sample Kolmogorov-Smirnov test. Quantitative data were presented as mean and standard deviation, and qualitative data as frequency and percentage. The linearity of the variables were assessed by using Pearson correlation analysis (r). All analyses were conducted by using the Statistical Package for Social Sciences (SPSS Inc., Chicago, IL) version 20.0 program. The statistical significance for all analyses was set at *p* < 0.05.

## Results

### Sample characteristics

A total of 147 patients including 78 girls (53.1%) and 69 boys (46.9%) were included in the study. Demographic characteristics of the patients are presented in Tables 1, 2 and 3. The mean age of the subjects was 8.63 ∓ 2.18, where 8.72 ∓ 2.37 in girls, and 8.52 ∓ 1.96 in boys. The mean VAS value was 5.5 ∓ 2.99 among girls and 5.52 ∓ 2.82 among boys.

**Table 1:**
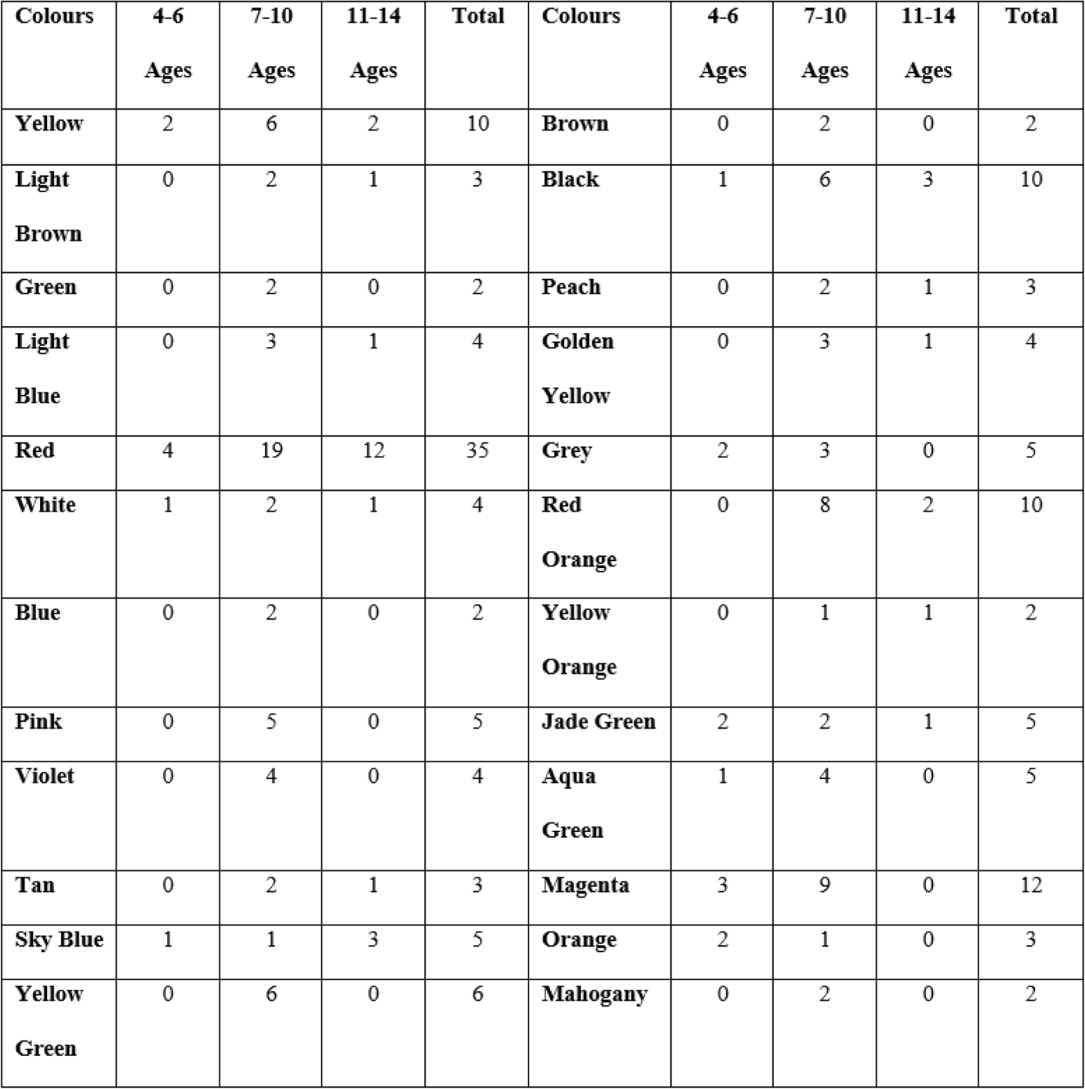
Frequency of colours in children with pain among age groups

**Table 2:**
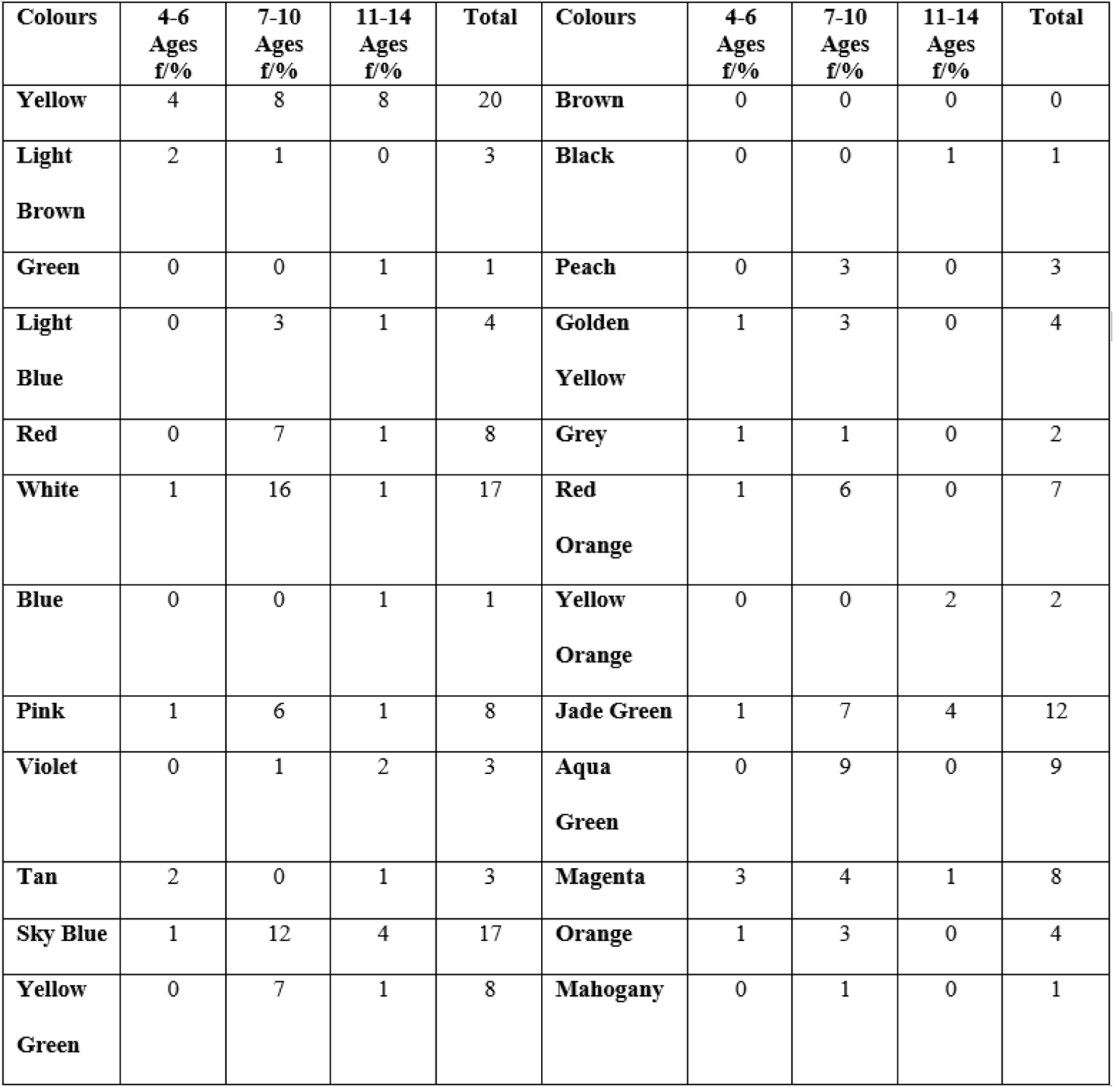
Frequency of colours in children without pain among age groups

**Table 3:**
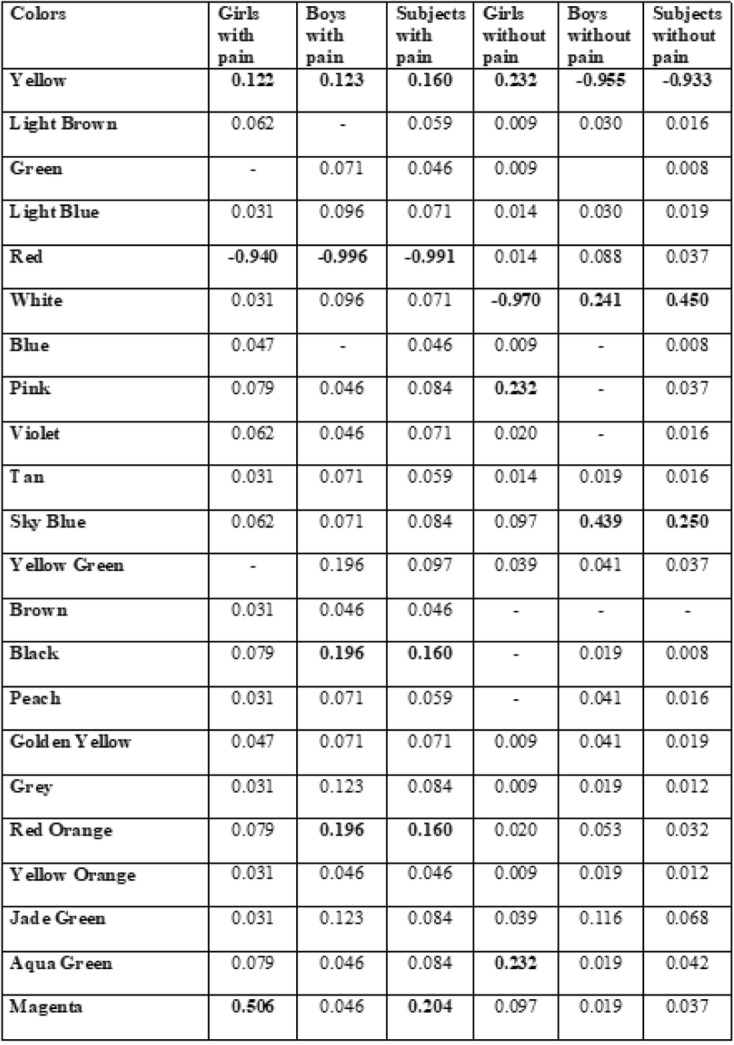
Factor analysis of colours in pain presence and absence in girls and boys

Highest factor loadings are indicated in bold

### Colour analysis

In the study, 14 colours in the presence of pain and 10 colours in the absence of pain were not used in the 4–6 aged group. All colours were chosen in the 7–10 age group in the pain presence (Table 1 and 2). Four different colours were not used in the 7–10 age group in the pain absence. Eleven and eight different colours were not chosen in the 11–14 age group in the pain presence and absence. When all age groups were evaluated, the presence of pain was mainly associated with red (*n* = 35), magenta (*n* = 12), black (*n* = 10), red orange (n = 10) and yellow (n = 10); and the absence of pain was associated with yellow (*n* = 20),white (*n* = 17), sky blue (17).

According to the gender, girls preferred red, magenta, and yellow in the presence of pain; while boys preferred red, black, red orange, yellow. In the absence of pain, the girls chose yellow, white,and pink while boys chose yellow, white, and sky blue (Table 3).

The principal component analysis showed that red has the highest factor loading in children with pain, whereas yellow was the other highest one in children without pain (Table 3).

### Visual analogue scale

There was no correlation between visual analogue scale (VAS) and pain presence (r = 0.096, p:0.245) and pain absence (r = 0.005, *p* = 0.948).

## Discussion

Parent-reports in the assessment of pain may cause incorrect pain management in children [19]. Incorrect pain management increases negative thoughts in children and causes reluctance to treatment; it has been reported that the parents feel helpless in connection with this [20]. Self-report in children is difficult, and an assessment pain tool is needed to recognize pain [5–7].

Children use colors as a kind of individual expression. Colored objects easily attract children [21]. Several studies focused on the selection of colours for measuring pain experiences in medicine (injections, venipuncture, intravenöz (IV) insertion and post-operative pain) [13–16]. However, little is known about using colours to assess pain in children with toothache [22]. Scott [23] reported that non-hospitalized children chose the red colour and warm colours (red, orange, yellow) from among nine colours to describe the needle injection and hammer operation. Savedra et al. [24] also found that hospitalized and non-hospitalized children (9–12 years old) exhibited a preference for the colour red and black, respectively. In a study of children hospitalized for children with musculoskeletal disease, red and purple were chosen to represent severe and moderate pain [25]. In a study, osteoarthritis (OA) pain was described using colours in adult patients [17]. The majority of participants chose red to describe high-intensity pain and chose white,yellow,orange,blue and green to describe the absence of pain.

In some studies, contrary to above information, children primarily selected black from among eight colours as the worst pain colour, and then they selected red and blue as the worst pain colours, respectively. Orange, the most common colour was selected for the least pain. A new scale, the colour circle pain scale (CCPS) in adult postoperative patient in Ghana, was consisted of six colours (white, green, gold, blue, red, black) [13]. Unbearable pain and the most severe pain were described with black and red; no pain was described with white. Arabic children in an American school described black and blue as the pain colour [26].

The present findings provide us with preliminary knowledge of the pain colour of Turkish children with toothache. The presence of pain was mainly associated with red, and the absence of pain was associated with yellow. Girls preferred red, magenta, and yellow in the presence of pain; while boys preferred red, black, red orange, and yellow. Red symbolizes that inflammation, emergency, fire, blood, anger and traffic lambs and has a strong visual impact on children [17]. In the absence of pain, the girls chose yellow, white, pink, and aqua green; while the boys chose yellow, white and sky blue. Interestingly, yellow colour was chosen both in pain absence and presence by girls and boys. Yellow is used for “no pain” meaning in many colour pain tools, since yellow is the brightest and most vibrant of colours, it often arouses optimism, joy and happiness in people. We think that the main reason for the association of pain with yellow colour is cultural perception. In Turkish language, yellow colour may be used negatively; as meaning pale, faded, and illness. Children in our study did not express the presence and absence of pain in green and blue.

Unlike other studies, colour choices were made from among 24 colours without making colour restriction. Nevertheless, accumulation was observed only in some colors. This accumulation shows that children make colour choices consciously. Gender and age are important factors in colour preference and use. Children in school period chose bright and vivid colours predominantly in the absence of pain. We think that the reason why preschool children (4–6 ages) used few colours is because of limited knowledge of colours. In pre-school period, more colour is preferred depending on the increase of colour knowledge. In the adolescent children (11–14 ages), the colour preferences are diminished and the presence and absence of pain are concentrated in certain shades of colour, indicating that progressive age groups can relate the pain feelings to colours more. The child in adolescence period can make more realistic choices thanks to what they learn and observe.

The VAS has been also used to examine children’s intensity of pain. In our study, there was no correlation between description of pain colour and responses on the VAS in Turkish children with toothache. The colour shows the presence of pain in children but does not give us full information about the pain intensity.

Since the verbal expression of feelings is an abstract phenomenon, it might continue to develop throughout the growth of children. The pain perception and expression, perhaps one of the the most subjective feelings in human, is strongly effected by gender, age, culture and so on. Instead of expecting a verbal expression from a child, a visual aid to express their pain on a color scale with a range of shades might help dentist to perceive the real feeling and the strength of pain in the clinical settings. In case the validation of color code with the children’s pain expression is established, color-aided pain communication in dentistry might reveal the nature and the perception of pain in children.

Limits of the study; We did not inquire about any past painful episodes in the children’s lives. In addition, we only studied children with acute pain so our results are not applicable to children with chronic pain. However, the suggestion should be taken with a word of caution, since the perception of pain is especially subjective in children. Therefore, clinical examination findings and appropriate radiological modalities should be used to make a prompt diagnosis. Further research for right pain management is required to explore the cultural pain perception profile.

## Conclusions

The relationship that children established with colours and concepts is an important field in which social, psychological and emotional dimensions need to be investigated. We think that the colours that children associate with pain feelings will increase the quality of communication between the patient and the physician. However, in order to associate pain with colours, a three-dimensional scale that varies according to societies, genders, and age should be established instead of one-dimensional standard colour scale.

## Abbreviations

CCPS: Colour circle pain scale
CHEOPS: Children’s Hospital of Easter of Ontario Pain Scale
IV: Intravenöz
OA: Osteoarthritis
SPSS: Statistical Package for Social Sciences
VAS: Visual analogue scale
